# Low back pain as an initial symptom of pregnancy‐associated breast cancer: a case report

**DOI:** 10.1186/s12905-021-01298-1

**Published:** 2021-04-15

**Authors:** Shunya Sugai, Eiko Sakata, Takumi Kurabayashi

**Affiliations:** 1 Department of Obstetrics and Gynaecology, Niigata City General Hospital, 463-7, Shumoku, Chuo-ku, Niigata, 950–1197 Japan; 2 Department of Breast Surgery, Niigata City General Hospital, 463-7, Shumoku, Chuo-ku, Niigata, 950-1197 Japan

**Keywords:** Low back pain, Breast cancer, Pregnancy, Compression fracture, Osteoporosis

## Abstract

**Background:**

Low back pain during pregnancy and postpartum is common and might not arouse clinical interest. Pregnancy-associated breast cancer is often found as a breast mass, but its diagnosis is difficult during pregnancy and postpartum. As more women delay their first pregnancies, its incidence may increase in the future.

**Case presentation:**

The patient was a 30-year-old gravida 3, para 3. She had low back pain from the second trimester of her previous two pregnancies, which improved spontaneously after delivery. In her third pregnancy, she again developed low back pain in the second trimester. Her delivery was normal. However, her low back pain continued for up to 7 months postpartum and then worsened sharply. A whole-body scan revealed a compression fracture due to multiple spinal metastases of breast cancer. As she had not complained about her breasts, they had not been closely examined.

**Conclusions:**

This case shows the importance of considering bone metastases from breast cancer in the differential diagnosis of patients with low back pain during pregnancy and postpartum.

## Background

Low back pain is common during pregnancy and postpartum and is usually caused by physiological changes associated with pregnancy. About half of low back pain cases associated with pregnancy resolve spontaneously within a year after delivery; therefore, it is widely considered a normal phenomenon [1].

Pregnancy-associated breast cancer (PABC) is breast cancer that occurs during pregnancy or within 12 months of delivery [2]. It is among the most frequently diagnosed cancers during pregnancy, with an incidence of approximately 1 in 3000 pregnant women. PABC usually initially presents with local symptoms, such as breast masses, and it is diagnosed using mammography and ultrasonography. Ultrasonography is particularly effective because it does not expose the fetus to radiation and can also be used when performing a percutaneous biopsy [3]. We treat PABC with surgery, chemotherapy, and radiotherapy. Other than radiotherapy, this can be provided during pregnancy, and pregnancy itself should not change the treatment [3]. Diagnoses of PABC are expected to increase in the future as more women opt to delay pregnancy [4]. However, changes in breast histology during pregnancy and postpartum make diagnosis more difficult, and delayed diagnosis is more common than in nonpregnant women [2]; as a result of these delayed diagnoses, PABC has a poor prognosis [4].

Here, we report a rare case of PABC with low back pain as the initial symptom. Diagnosing PABC was problematic because the main complaint was low back pain, which often occurs during pregnancy and postpartum.

## Case presentation

A 30-year-old gravida 3, para 3 presented with low back pain, seven months after her third delivery.

Her first two deliveries were normal vaginal deliveries without any problems. In both pregnancies, low back pain occurred from the second trimester, and spontaneously improved after delivery. She was naturally healthy with no history of metabolic bone disease, menstrual abnormalities, previous fractures, or eating disorders. She also had no reported family history of osteoporosis or malignancy.

During her third pregnancy, she again became aware of low back pain from the second trimester. She had no problems with walking or activities of daily living. As it was similar to the lower back pain of previous pregnancies, it was thought to be pregnancy-associated low back pain and was not specifically examined. The pregnancy course was good, with a normal vaginal delivery at term. Her baby was healthy.

**Fig. 1:**
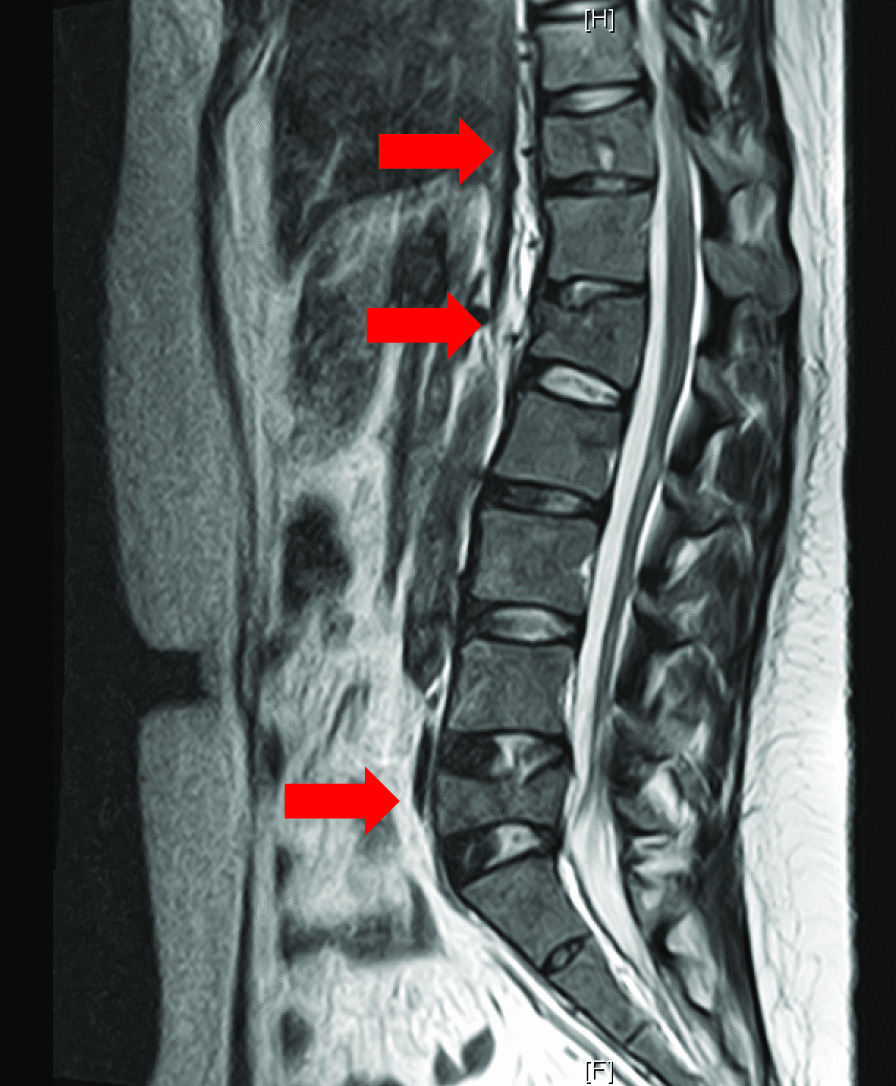
Thoracolumbar MRI T2 image. Multiple compression fractures at Th11, L1 and L5 (arrows)

However, her low back pain persisted after 5 months postpartum. She visited an orthopaedic clinic and was treated conservatively with analgesics. Her low back pain worsened sharply 7 months after delivery, so she went to another orthopaedic clinic. She had no episodes of trauma. A spinal X-ray and MRI were taken to rule out spinal lesions. Multiple compression fractures were observed at Th11, L1 and L5 **(**Fig. 1**)**. She was referred to our hospital to investigate the cause.

The patient was 162 cm tall, weighed 69 kg, and had a body mass index of 26 when she visited our hospital. The pain was localized to the mid-lumbar region above the sacrum and was accompanied by tenderness. She had difficulty walking due to back pain and used a wheelchair at the time of her visit. The pain was exacerbated by flexion and activity and was partially relieved by rest. Analgesics had limited effect. No sensory or movement impairment was observed in her lower limbs. She continued to breastfeed. Both breasts were rather firm, which was thought to be attributable to the breastfeeding period. No difference between the left and right breasts was observed, and the patient reported no changes in skin (e.g., indentations), secretions other than milk, or pain in her breasts.

We suspected pregnancy- and lactation-associated osteoporosis (PLO) as the cause of the multiple compression vertebral fractures. Dual-energy X-ray absorptiometry revealed a bone mineral density (BMD) of 0.854 g/cm^2^, T-score: −1.4 for the lumbar spine (L2–L4), and BMD: 0.801 g/cm^2^, T-score: 0.2 for the femoral neck, which are within normal ranges.

**Fig. 2:**
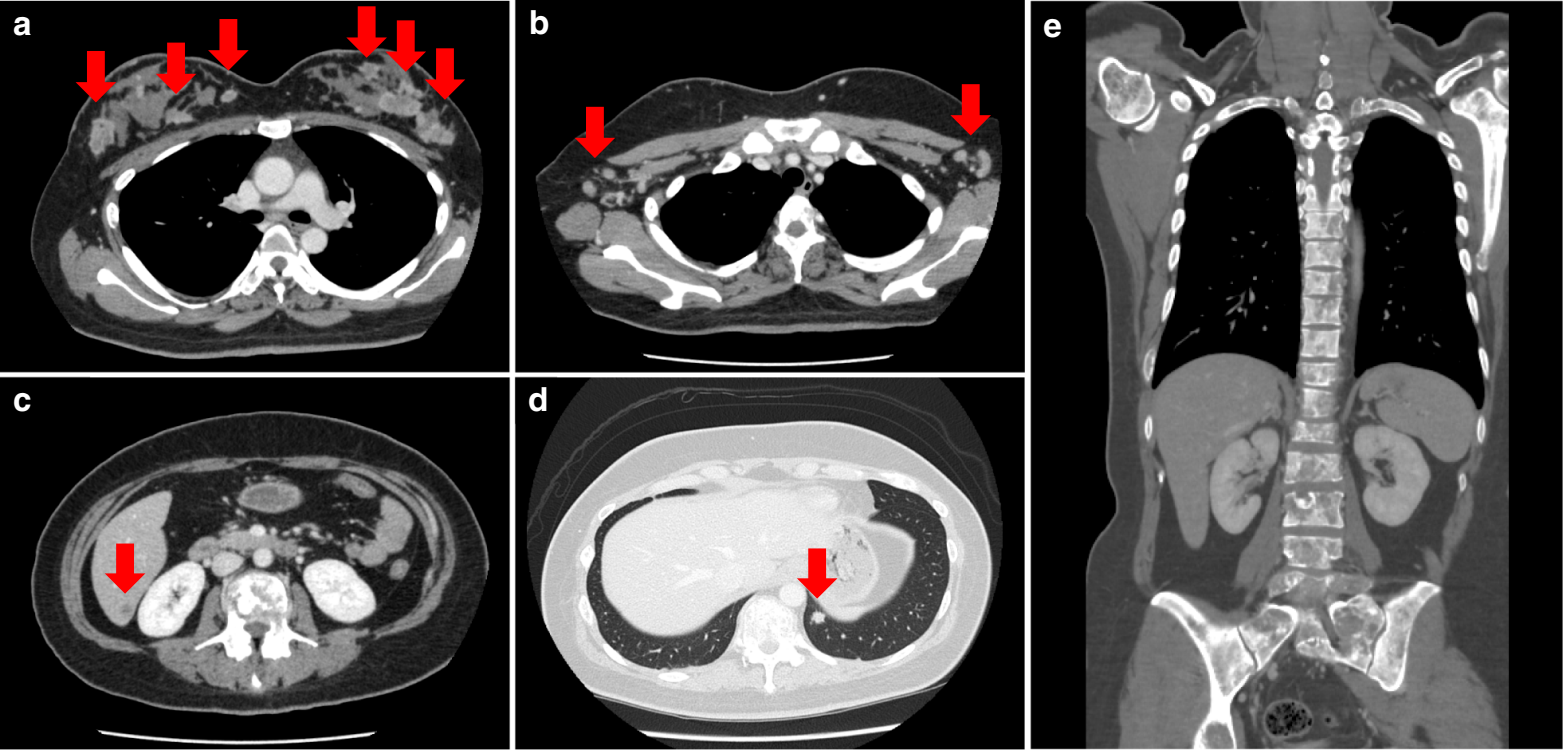
Contrast-enhanced CT images. **a** Contrast-enhanced masses occur frequently in bilateral breasts (arrows). **b** Metastasis to bilateral axillary lymph nodes (arrows). **c** Metastasis in the S7 area of the liver (arrow). **d** Metastasis to the lower lobe of the left lung (arrow). **e** Metastasis on all observable vertebral bodies

**Fig. 3:**
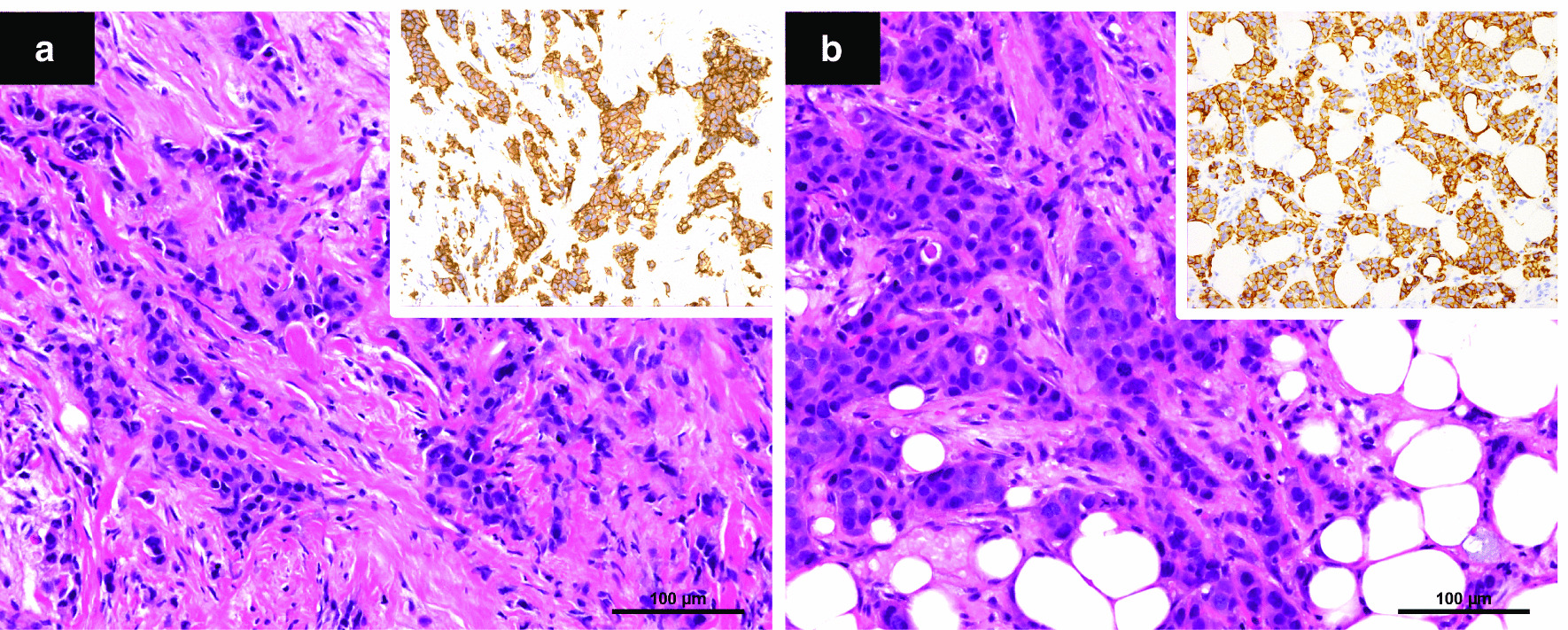
Bilateral invasive ductal carcinoma; histopathological findings. **a** Right breast. **b** Left breast. Haematoxylin & eosin, original magnification × 20. Insets: HER2, 3+

Blood test results are shown in Table 1. They indicated hepatic dysfunction and elevated serum calcium. The bone resorption marker was high. We decided to perform systemic CT because of her unexplained hepatic dysfunction. It showed multiple contrast-enhanced masses in both breasts, multiple lymph node metastases, multiple bone metastases in the spine, lung metastasis, and liver metastasis **(**Fig. 2**)**. We suspected advanced breast cancer. Bilateral needle biopsies of her breast tissue revealed invasive HER2-positive ductal carcinoma **(**Fig. 3**)**. Her low back pain was caused by a compression fracture from spinal metastasis of breast cancer. She started chemotherapy with paclitaxel and trastuzumab at 8 months postpartum. Two and a half years after the start of treatment, she is in complete clinical remission.

**Table 1:** Blood test results

Laboratory data		
*Complete blood count*		
WBC	7290	/µL
RBC	4.74	× 10^6^/µL
Hb	13.7	g/dL
Hct	40.9	%
Plt	185	× 10^4^/µL
*Blood biochemical test*		
TP	7.9	g/dL
Alb	4.4	g/dL
T-Bil	0.6	mg/dL
AST	200	IU/L
ALT	273	IU/L
ALP	852	IU/L
LDH	1041	IU/L
γ-GTP	381	IU/L
CK	82	IU/L
BUN	20.7	mg/dL
Cre	0.74	mg/dL
Na	137	mmol/L
K	4.1	mmol/L
Cl	101	mmol/L
Ca	10.5	mg/dL
CRP	2.12	mg/dL
NTX	65.2	nmolBCE/L

*Alb* albumin, *ALP* alkaline phosphatase, *ALT* alanine aminotransferase, *AST* aspartate aminotransferase, *BUN* blood urea nitrogen, *Ca* calcium, *CK* creatine kinase, *Cl* chlorine, *Cre* creatinine, *CRP* C-reactive protein, *γ-GTP* γ-glutamyl transpeptidase, *Hb* haemoglobin, *Hct* haematocrit, *K* potassium, *LDH* lactate dehydrogenase, *Na* sodium, *NTX* N-terminal crosslinking telopeptide of type 1 collagen, *Plt* platelets, *RBC* red blood cells, *T-bil* total bilirubin, *TP* total protein, *WBC* white blood cells

## Discussion

In this article, we present a patient whose pregnancy-associated breast cancer (PABC) was difficult to diagnose because it initially presented with low back pain.

Low back pain in pregnancy and postpartum is common and is associated with several physiological factors. First, joint relaxation due to elevated levels of relaxin, progesterone, and oestrogens causes back pain. Second, the dilation of the uterus stretches and weakens the abdominal muscles, and the centre of gravity moves forward, stressing the lumbar muscles. Third, the axial load on the spine compresses the disc. Finally, compression of both the aorta and vena cava by the uterus can lead to low back pain by impairing the metabolism of neural structures due to local reduced oxygen saturation and venous congestion [1].

About 50 % of pregnant women experience low back pain [5], and about 10 % have persistent pain that lasts for about 2 years [6]. Low back pain in pregnancy and postpartum might be considered a normal phenomenon that requires no special attention. A history of low back pain during a previous pregnancy is a strong predictor, with an 85 % chance of repeating [7]. Our patient also had low back pain during her previous pregnancies, and the low back pain in this pregnancy had the same characteristics, until 7 months postpartum, when the situation worsened; even then, the increase in discomfort was mild, so it was considered to be a normal phenomenon.

Low back pain in pregnancy and postpartum has a diverse differential diagnosis. We believe that a thorough medical history and physical examination can guide diagnosis [1].

PLO was first reported by Nordin and Roper in 1955 [8]. PLO is a rare form of osteoporosis for which the exact pathophysiological mechanism remains unknown. Although its estimated incidence is 0.4 per 100,000 women, it may be underestimated [9]. Most cases of PLO develop early after childbirth from the third trimester, and the main symptoms are severe low back pain and stature reduction due to multiple compression fractures [10]. Our patient had multiple vertebral fractures. We strongly suspected PLO. However, PLO was ruled out because the BMD was normal.

PABC has attracted increasing attention in recent years. Among women with breast cancer, PABC occurs in 2.6–6.9 % of those aged 45 years or younger, and in up to 15.6 % of women under the age of 35 [11]. The risk of PABC has been shown to be higher in women over the age of 35 in their first pregnancies, and this risk remains high for the next 5 years [12]. Therefore, PABC rates may increase in the future as more women delay their first pregnancies. Baseline breast examination may be recommended in the early stages of pregnancy.

During pregnancy and lactation, hyperplasia of normal linear and vascular structures causes the breasts to become dense, and small masses may be felt. Therefore, our level of clinical suspicion of breast cancer is low. As a result, the diagnosis of PABC can be delayed by up to 13 months [11]. In contrast, in non-pregnant women, breast cancer is usually diagnosed within a month after the mass is detected [13]. 21 % of PABCs are found in Stage I or II, compared with 54 % in non-pregnant women [4]. Because of this delay in diagnosis, the prognosis for PABC is generally poor.

Breast masses are the most common initial symptom of PABC. Wang et al. [14] reported that in their study of 142 women with PABC, the initial symptoms in all 142 patients were breast masses or papillary discharges. We found no reports of low back pain as the initial symptom of PABC. Our patient’s initial symptom was low back pain, which is an ordinary phenomenon during pregnancy and postpartum. Therefore, we may have had difficulty diagnosing PABC.

In conclusion, bone metastases from breast cancer may be a cause of low back pain during pregnancy and postpartum. As PABC is expected to become more common in the future because of the increasing age of women during pregnancy, and as delayed diagnosis can lead to poor prognosis, a higher index of suspicion is warranted for PABC in these patients.

## Data Availability

All data related to this case report are available from the corresponding author on reasonable request.

## Abbreviations

BMD: Bone mineral density
CT: Computed tomography
HER2: Human epidermal growth factor receptor type 2
MRI: Magnetic resonance imaging
PABC: Pregnancy-associated breast cancer
PLO: Pregnancy and lactation associated osteoporosis
